# Characteristics of long COVID in patients with autoimmune rheumatic diseases: a systematic review and meta-analysis

**DOI:** 10.1093/rap/rkae027

**Published:** 2024-03-02

**Authors:** Der-Yuan Chen, Po-I Huang, Kuo-Tung Tang

**Affiliations:** Rheumatology and Immunology Center, China Medical University Hospital, Taichung, Taiwan; College of Medicine, China Medical University, Taichung, Taiwan; College of Medicine, National Chung Hsing University, Taichung, Taiwan; Department of Internal Medicine, Taichung Veterans General Hospital, Taichung, Taiwan; Division of Allergy, Immunology, and Rheumatology, Taichung Veterans General Hospital, Taichung, Taiwan; Faculty of Medicine, National Yang Ming Chiao Tung University, Taipei, Taiwan; Ph.D. Program in Translational Medicine and Rong Hsing Research Center for Translational Medicine, National Chung Hsing University, Taichung, Taiwan

**Keywords:** fibromyalgia, long COVID, rheumatic disease, systemic autoimmune rheumatic disease

## Abstract

**Objectives:**

Numerous cases of long coronavirus disease (long COVID) have been reported in patients with autoimmune rheumatic diseases (ARDs). Despite the reviews on clinical manifestations of long COVID in the general population, systematic reviews on ARD patients are scarce. Herein, we conducted a systematic review and meta-analysis on the prevalence and characteristics of long COVID in ARD patients.

**Methods:**

We searched the literature in PubMed and Embase as of 27 December 2022. Cohort, cross-sectional and case–control studies relevant to long COVID in ARD patients were collected. Stratification based on the severity of COVID infection and subtypes of rheumatic diseases [systemic autoimmune rheumatic disease (SARD) *vs* non-autoimmune rheumatic disease (NARD)] was also undertaken. A random-effects model was used in the meta-analysis.

**Results:**

A total of 15 relevant studies were identified from the literature. The prevalence of long COVID was 56% (95% CI 34, 76) in 2995 patients. Hospitalized COVID patients had a higher proportion of long COVID than non-hospitalized patients. The prevalence of long COVID was similar between SARD and NARD patients. In terms of symptoms, fatigue, arthralgia and pain were commonly reported in long COVID patients with ARDs.

**Conclusion:**

The characteristics of long COVID in ARD patients are generally similar to those in the general population despite a higher prevalence and a higher proportion of arthralgia and pain.

Key messages

- Studies on clinical manifestations of long COVID in rheumatic patients are relatively few.
- Long COVID is more prevalent and associated with more arthralgia and pain in rheumatic patients.
- Vaccination and timely treatment for COVID in rheumatic patients may prevent long COVID.

## Introduction

The coronavirus disease 2019 (COVID-19) pandemic has been a great challenge worldwide over the last 3 years. Its clinical manifestations involve multiple organs and include fever, cough, shortness of breath, sore throat, myalgia, arthralgia, headache, rhinorrhoea, nausea, vomiting, diarrhoea and olfactory and gustatory dysfunctions [1, 2]. Despite the heterogeneity of symptoms between individuals and disease severity assessment across studies [3, 4], most patients were asymptomatic or presented with only mild symptoms. Some patients suffered from severe symptoms, with even life-threatening complications. Notably, a small proportion of COVID-19 patients developed persistent residual symptoms months after recovery from infections of severe acute respiratory syndrome coronavirus 2 (SARS-CoV-2). Such a phenomenon is known as either long COVID, post-COVID syndrome or post–acute COVID-19 sequelae [5]. A number of studies have shown that long COVID is prevalent in COVID-19 patients [6]. The persistent symptoms are variable and they involve organs such as the heart, brain, spleen, liver, gastrointestinal tract, kidney, pancreas and lung [7]. Currently, no definition of long COVID exists with consensus, mainly due to the heterogeneity of its symptoms [8]. Nevertheless, both the persistent and the new-onset symptoms after acute SARS-CoV-2 infection are included. The time points at which symptoms are assessed after the COVID-19 infection vary; e.g. for the Centers for Disease Control and Prevention (CDC), the time is at least 4 weeks later; for the World Health Organization (WHO) and National Institute for Health and Care Excellence (NICE), it is at least 12 weeks later [8].

The pathogenesis of long COVID remains unclear. The role of the immune system has been implicated [5]. Recently, the results of large cohort studies reported a higher risk of developing systemic autoimmune rheumatic disease (SARD) in COVID-19 patients, which suggested a persistent immune dysregulation long after resolution of COVID-19 infection [9, 10]. Both the disease severity and prognosis after SARS-CoV-2 infection are worse in SARD patients [11]. However, relevant studies regarding long COVID in these patients are scarce. Furthermore, clinical features of long COVID syndrome may be modified by the use of corticosteroids and immunosuppressants in patients with autoimmune rheumatic diseases (ARDs). In this systematic review, we aimed to summarize the characteristics of long COVID in ARD patients.

## Materials and methods

### Literature search

**Figure 1.**
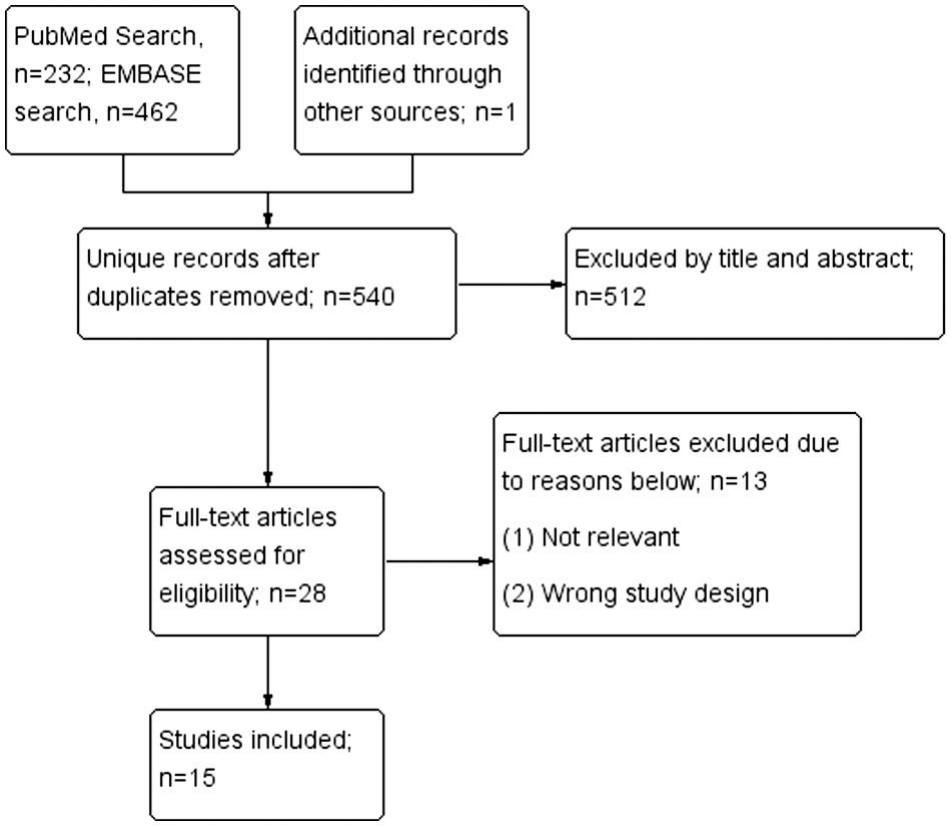
The algorithm of the literature search

We focused on studies investigating long COVID syndrome in ARD patients. We searched both the Embase and MEDLINE databases and reviewed literature through 27 December 2022, irrespective of the language of publication. The search strategy is illustrated in Supplementary Table S1, available at *Rheumatology Advances in Practice* online, combining two sets of terms: one set involved a variety of ARDs and the other involved long COVID. A total of 15 studies were identified (Fig. 1) [12–26]. This systematic review was registered in PROSPERO (CRD42023451768).

### Selection of studies

All three authors (K.T.T., P.I.H. and D.Y.C.) independently assessed the titles and abstracts identified. The relevant full-text articles were then retrieved. Two authors (K.T.T. and P.I.H.) independently evaluated the full-text articles for eligibility, i.e. articles on long COVID in ARD patients. We included observational studies such as case–control, cross-sectional and cohort studies. Conference abstracts were also included, but preprints, case reports and case series were excluded. Controversy, if present, was resolved through group discussion.

### Data extraction

Information regarding the prevalence in ARD patients was retrieved and recorded in a standardized Excel file (Microsoft, Redmond, WA, USA). The outcome measures included the prevalence of long COVID and its symptoms. The risk factors for long COVID development were also documented and represented as odds ratios (ORs).

### Risk of bias

Risk of bias was evaluated based on the Risk of Bias in Non-Randomised Studies of Interventions (ROBINS-I) [27, 28]. This tool incorporates seven major domains, including confounding, selection of participants, classification of intervention, deviation from interventions, missing outcome data, measurement of outcomes and selection of the reported result. The overall risk of bias was rated as 0, no information; 1, low risk; 2, moderate risk; 3, serious risk; and 4, critical risk. Two authors (P.I.H. and K.T.T.) independently assessed these risks of bias. Any disagreement, if present, was resolved through group discussion.

### Data synthesis and statistical analyses

We summarized the prevalence of long COVID in all ARD patients. Stratification was made based on the severity of COVID-19 infection and subtypes of rheumatic diseases [SARD *vs* non-autoimmune rheumatic disease (NARD)]. A random-effects model was used in the meta-analysis based on the procedure of DerSimonian and Laird [29]. Heterogeneity was quantified using τ^2^, χ^2^ and *I*^2^ as measured in the Mantel–Haenszel model. Funnel plots, as well as Begg’s and Egger’s tests, were used to evaluate the publication bias if more than two studies were included in the meta-analyses. All statistical analyses were performed using Stata version 14.0 (StataCorp, College Station, TX, USA).

## Results

### Study characteristics

Characteristics of the enrolled studies are shown in Supplementary Table S2, available at *Rheumatology Advances in Practice* online. Most study participants were adult female Caucasians in Western countries. The mean/median age of study participants ranged from 47 to 71 years. Most studies involved SARD patients. The most common SARD was RA and the most common NARD was FM. Most of the studies surveyed long COVID symptoms based on clinical evaluation, with these persistent symptoms lasting for a minimum of 28 days after COVID-19 infection.

### Prevalence of long COVID in ARD patients

**Figure 2.**
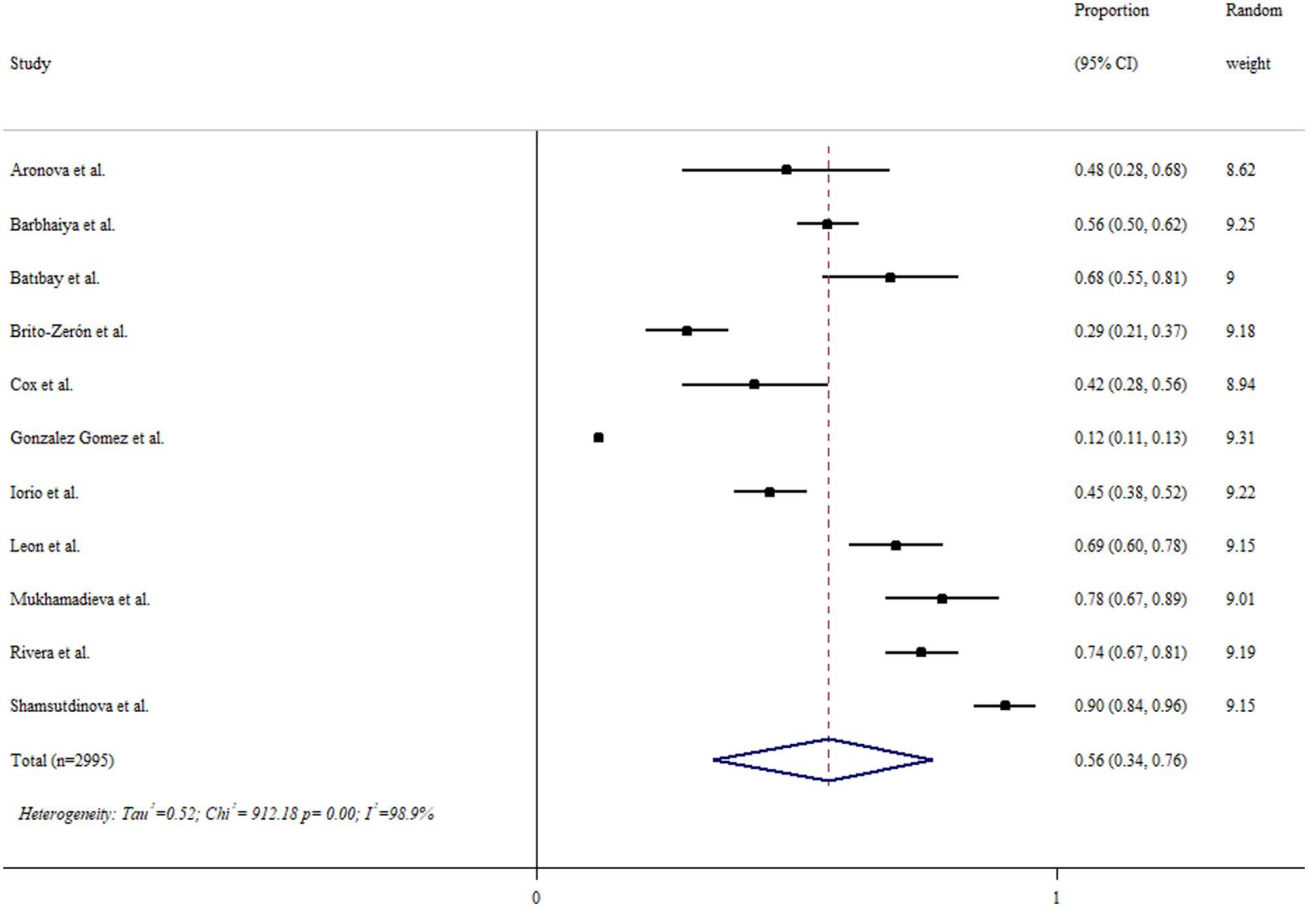
The prevalence of long COVID in patients with rheumatic diseases. The black squares represent the effect estimates of the individual studies and the diamonds represent the summary effect estimates

**Figure 3.**
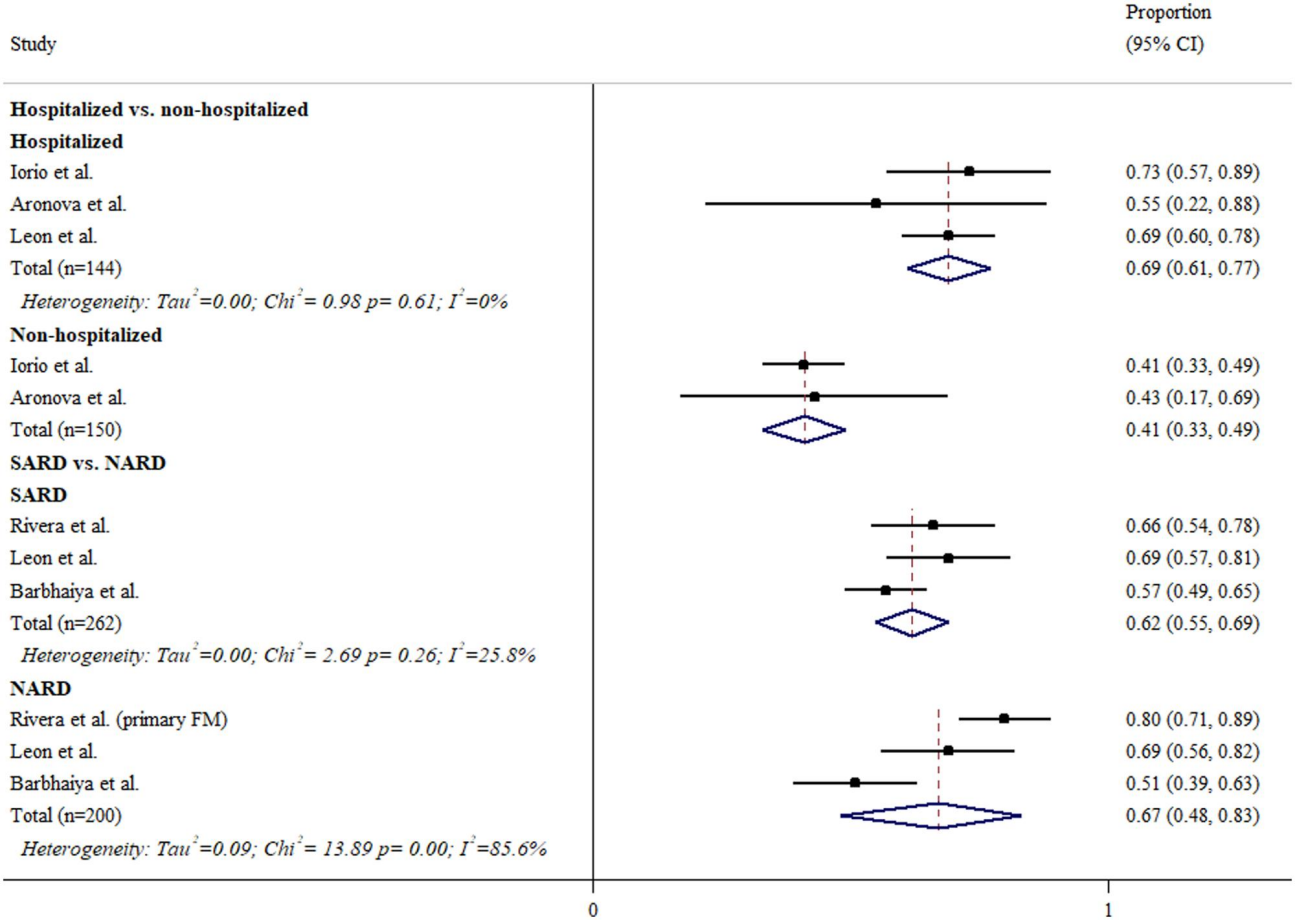
The prevalence of long COVID in rheumatic patients after stratification by hospitalization status and disease subtypes. The black squares represent the effect estimates of the individual studies and the diamonds represent the summary effect estimates

As illustrated in Fig. 2, the prevalence of long COVID in SARS-CoV-2-infected ARD patients was 56% (95% CI 34, 76). Its prevalence appeared higher in hospitalized COVID-19 patients when compared with non-hospitalized patients [69% (95% CI 61, 77) *vs* 41% (95% CI 33, 49)] (Fig. 3). There was no difference in the prevalence rates of long COVID between SARD and NARD patients [62% (95% CI 55, 69] *vs* 67% (95% CI 48, 83)].

### Symptoms of long COVID in ARD patients

**Figure 4.**
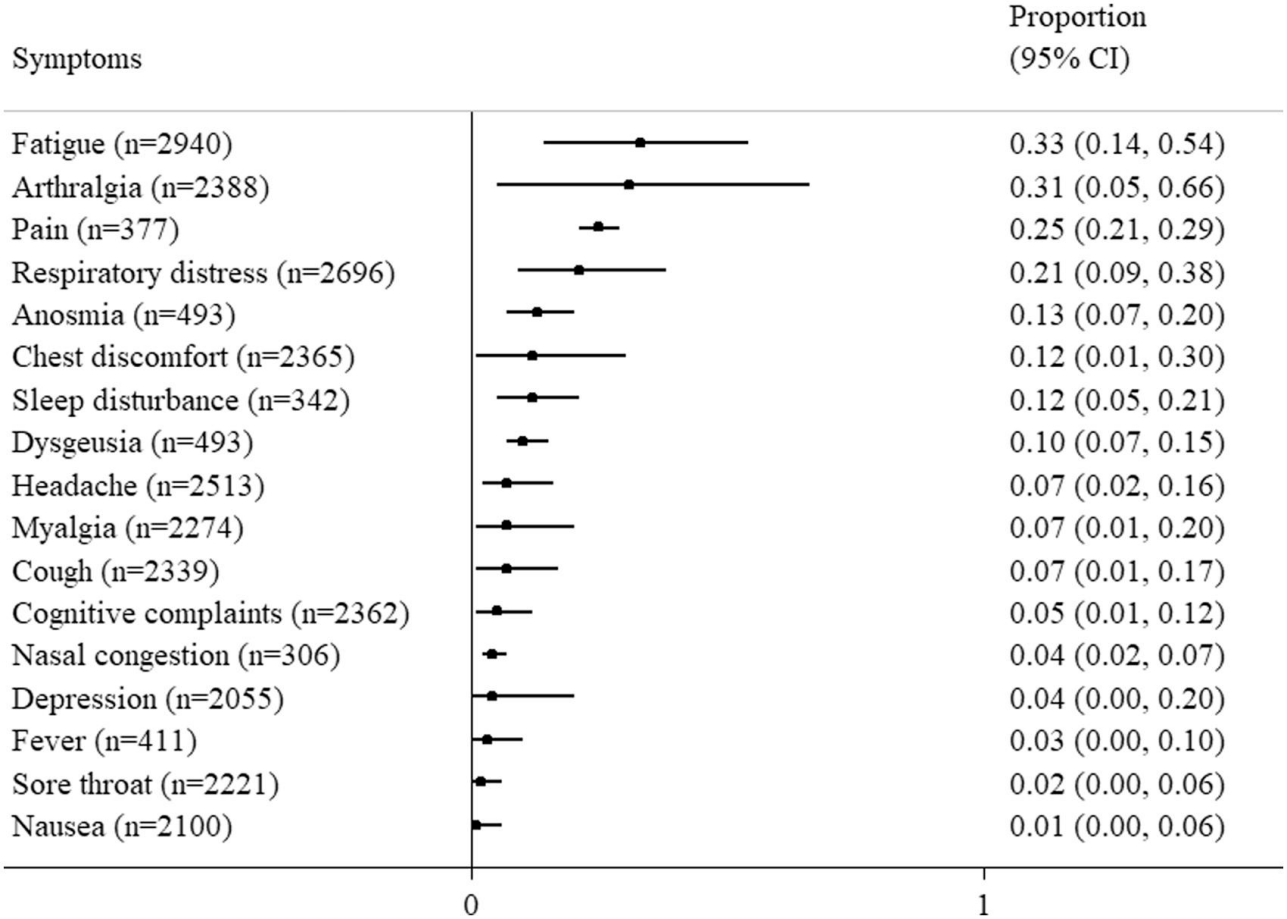
The prevalence of long COVID symptoms in patients with rheumatic diseases. The black squares represent the summary effect estimates.

As shown in Fig. 4, fatigue was reported as the most common symptom and its prevalence was 33% (95% CI 14, 54). Arthralgia and pain were the second and third most common symptoms. The prevalence of arthralgia was 31% (95% CI 5, 66) and the prevalence of pain was 25% (95% CI 21, 29). Respiratory distress was also common and its prevalence was 21% (95% CI 9, 38). One, two or three long COVID symptoms were found in 20–89%, 35–61% and 39–76%, respectively, in ARD patients with long COVID [14, 15, 19, 22, 23, 26]. In SARD patients in the Danish national health registry hospitalized due to COVID-19 infection, Nogard *et al.* reported higher risks for later hospitalization due to respiratory diseases [adjusted hazard ratio (HR) 1.20 (95% CI 1.02, 1.58)] and infections [adjusted HR 1.55 (95% CI 1.26, 1.92)] [24]. In rheumatic patients with long COVID, Barbhaiya *et al.* [13] observed worse anxiety and depression based on the Patient-Reported Outcomes Measurement Information System (PROMIS) when compared with patients without long COVID. Cox *et al.* [17] conducted a survey using the 36-item Short-Form in COVID-19-infected rheumatic patients and found that mental components, but not physical components, were worse in those patients who developed long COVID *vs* those who did not.

### Potential risk factors for long COVID in ARD patients

As shown in Table 1, the severity of COVID-19 infection (hospitalization, pneumonia, symptoms severity, length of hospitalization/intensive care unit stay) was associated with the development of long COVID in ARD patients. Notably, hospitalization due to COVID-19 was associated with long COVID development, with an OR of 3.54–8.29 [15, 21]. Gomez *et al.* [19] previously reported a higher risk for long COVID in non-Caucasians. In terms of disease activity, Gomez *et al.* [19] reported no difference in the proportion of low disease activity/remission between those patients who developed and those who did not develop long COVID. Aronova *et al.* [12] also observed no difference in disease activity of RA between those patients who developed and those who did not develop long COVID. In terms of the prescribed medications, the use of hydroxychloroquine during COVID-19 infection was positively associated with the development of long COVID [OR 2.52 (95% CI 1.00, 6.47)]. Prior use of systemic corticosteroids and cyclophosphamide was similarly associated with long COVID [OR 4.95 (95% CI 1.65, 14.82) and 11.35 (95% CI 1.56, 112.97), respectively].

**Table 1.** Risk factors for long COVID in rheumatic patients

Study	Risk factors
Barbhaiya *et al.* [13]	Comorbidities [OR 3.17 (95% CI 1.89, 5.32)], smoking [OR 2.21 (95% CI 1.19, 4.09)], corticosteroids use [OR 4.95 (95% CI 1.65, 14.82)] and COVID-19 symptoms
Batıbay *et al.* [14]	Non-smoking [OR 7.70 (95% CI 1.67, 35.43)]
Brito-Zerón *et al.* [15]	Elevated CRP [OR 8.60 (95% CI 1.33, 104.44)], hydroxychloroquine use [OR 2.52 (95% CI 1.00, 6.47)] and hospital admission [OR 4.96 (95% CI 1.91, 13.87)]
Gomez *et al.* [19]	Non-Caucasian [OR 1.44 (95% CI 1.07, 1.95)], years of education [OR 1.05 (95% CI 1.00, 1.09)], cyclophosphamide [OR 11.35 (95% CI 1.56, 112.97)], symptoms of COVID-19 [OR 13.26 (95% CI 2.75, 242.08)], severe COVID-19 disease [OR 2.46 (95% CI 1.68, 3.57)] and intensive care unit hospitalization days [OR 1.09 (95% CI 1.05, 1.14)]
Husni *et al.* [20]	Better pre-COVID patient-reported outcomes
De Iorio *et al.* [21]	Hospitalization for COVID-19 [OR 3.54 (95% CI 1.27, 9.87)] and initial COVID-19 symptom count [OR 1.38 (95% CI 1.17, 1.63)]
Leon *et al.* [22]	Female sex [OR 3.53 (95% CI 1.24, 10.08)], age >60 years (OR 0.09–0.11), comorbidities [OR 1.90 (95% CI 1.09, 3.31)], lymphopenia [OR 3.17 (95% CI 1.07, 9.40)] and pneumonia [OR 10.1 (95% CI 1.50, 66.01)]

### Long COVID in FM patients

Büyükşireci *et al.* [16] compared 77 FM patients recovering from COVID-19 infection, irrespective of the presence of long COVID, and 57 FM patients who did not contract COVID-19. They found that COVID-19-infected FM patients had similar disease severity, but had higher HADS anxiety scores and widespread pain index when compared with patients who did not contract COVID-19. Rivera *et al.* [21] compared 78 FM patients and 56 SARD patients after COVID-19 infection and found that FM patients reported more long COVID symptoms and worse patient global assessment compared with SARD patients.

### Risk of bias

As shown in Supplementary Fig. S1, available at *Rheumatology Advances in Practice* online, 5 (33%) of the 15 studies were recognized as having a serious risk of bias.

### Publication bias

We demonstrated the existence of potential publication bias with regards to the prevalence of long COVID and its symptoms in rheumatic patients except for anosmia (Supplementary Figs S2 and S3, available at *Rheumatology Advances in Practice* online).

## Discussion

In this systematic review and meta-analysis, we found a higher prevalence of long COVID in ARD patients when compared with the general population. In addition, the proportion of arthralgia and pain appeared higher in ARD patients with long COVID. The prevalence of long COVID was similar between SARD and NARD patients. Disease severity of acute COVID-19 infection was associated with an increased risk for long COVID in ARD patients. Despite the limited number of studies, the use of medications might affect such risk in these patients.

Several factors have been implicated in the generation of long COVID, including viral persistence, tissue damage, metabolic change, autonomic dysfunction and psychosocial burden [30–33]. SARS-CoV-2 infection could promote the production of a myriad of cytokines and chemokines, the so-called cytokine storm, which likely leads to autoimmune and rheumatic manifestations [34]. The immune system is probably involved in the pathogenesis of long COVID. Previous reports of long COVID patients showed elevated levels of type I and type III IFNs, TNF-α, G-CSF, IL-17A, IL-6, IL-1β and IL-13, whereas IFN-γ-induced protein 10 (IP-10) levels decreased, when compared with levels in the acute phase of COVID-19 infection [35, 36]. Long COVID patients also had activated innate immune cells such as myeloid cells, Th9, CD4^+^ effector memory T cells, CD8^+^ effector T cells and naïve B cells [35, 36]. Furthermore, persistently elevated levels of anti-SARS-CoV-2 IgG antibodies were noted in these patients [35]. Interestingly, a murine experiment showed neuroinflammation after SARS-CoV-2 infection and, in particular, CCL11 persistently impaired neurogenesis and glial activity [5]. Consistent with this, circulating levels of CCL11 are elevated in long COVID patients with cognitive symptoms.

Owing to global efforts, the occurrence of devastating complications of acute COVID-19 infection, such as sepsis, respiratory failure and even death, have decreased. However, long-term sequelae of COVID-19 infection emerged [8]. Long COVID comprises >100 symptoms and significantly impairs patients’ quality of life. It is the next conundrum we need to face in the post-pandemic era. SARD patients have a more severe disease course and higher mortality during acute COVID infection when compared with the general population. Moreover, the use of Janus kinase inhibitors and rituximab was associated with severe COVID-19 in patients with RA [37]. We hypothesized that ARD patients are predisposed to long COVID due to pre-existing immune dysregulation and therefore show more severe symptoms with acute COVID-19 infections. A prior meta-analysis estimated the prevalence of long COVID in the general population at 43% (95% CI 0.39, 0.46) [38]. We noted a higher prevalence of long COVID among ARD patients despite heterogeneity across studies. On the other hand, Fernández de las Peñas *et al.* [18] conducted a multicentre study in Spain based on telephone interviews of 1969 participants. They found that pre-existing rheumatic diseases are not independently associated with the development of long COVID [OR 1.46 (95% CI 0.89, 2.40)]. In addition, SARD patients, who had an inherently exaggerated immune response, are not more likely to develop long COVID than NARD patients. More cohort studies are needed to clarify this issue.

In terms of symptoms of long COVID, fatigue was most commonly found. This is consistent with results in the general population [38]. In the study of Brito-Zerón *et al.* [15], >90% of SARD patients with long COVID have fatigue. Pain-related symptoms were the second most common symptom in ARD patients with long COVID, which was different from that in the general population (memory problems) [38]. This is probably due to the inherent pain susceptibility in ARD patients [39].

We found that the prevalence of long COVID in ARD patients with hospitalization due to COVID-19 was higher than that in patients without hospitalization. This finding is consistent with that in the general population [38]. COVID-19 severity, as measured by different parameters, is also a potential risk factor based on studies of ARD patients. It is therefore possible to alleviate the severity of acute COVID-19 infection to reduce the occurrence of long COVID. The prevalence of long COVID was slightly higher in Asian countries than in Europe and the USA [38]. Interestingly, non-Caucasian ethnicity was associated with a higher risk for long COVID [OR 1.44 (95% CI 1.07, 1.95)] in ARD patients, as reported by Gomez et al. [19]. In terms of medications, the limited data suggest a potential influence of hydroxychloroquine, corticosteroids and cyclophosphamide on the risk for long COVID in ARD patients. Such associations may be confounded and further studies of larger cohorts are needed.

FM patients had a similar symptom profile with long COVID [40]. In addition, viral illness has been reported to cause FM syndromes [41]. Two studies investigated long COVID in FM patients. Rivera *et al.* [25] found a trend of a higher percentage of long COVID in FM patients when compared with SARD patients (80% *vs* 66%; *P* = 0.081). As expected, FM patients reported more long COVID symptoms than SARD patients, likely related to the similarity of symptoms between FM and long COVID. However, they did not find an increased severity of FM, as measured by the Polysymptomatic Distress Scale, after COVID-19 infection, although a previous report showed increased FM severity during acute COVID infection [42]. Büyükşireci *et al.* [16] found no difference in disease severity, except for anxiety, between FM patients with and without prior COVID-19 infection. These findings do not corroborate the thesis that FM and long COVID share similar pathogenic mechanisms.

Currently there is no effective treatment for long COVID. The National Institutes of Health recently launched trials on the Researching COVID to Enhance Recovery initiative. Across the USA, trial interventions included a longer dosing of antiviral agents (nirmatrelvir and ritonavir), cognitive training and transcranial direct current stimulation. Results of these trials will likely shed light on the treatment options for long COVID. In terms of preventive measures, vaccination and antiviral agents have been shown to be associated with a lower risk for development of long COVID in cohort studies, although little is known on ARD patients [43, 44].

Our review has some limitations. First, the baseline demographics of study populations, their comorbidities, variants of the SARS-CoV-2 virus [45] and outcome definitions varied considerably across studies. Such heterogeneity in the study population makes interpretation of the results difficult. For instance, the *I*^2^ statistic was 98.9% in terms of the meta-analysis of the prevalence of long COVID-19 in ARD patients. Second, underrepresentation of other ethnic groups such as Asians, and age groups such as adolescents and children, is obviously a knowledge gap that requires more data. Lastly, the relatively few studies with a small sample size and significant risk of bias suggested an urgent need to conduct more research to elucidate the question in ARD patients, who are potentially susceptible to the development of long COVID. We performed analyses after excluding studies with a serious risk of bias. The results changed slightly and the prevalence of long COVID was even higher [65% (95% CI 48, 81)] in ARD patients (Supplementary Fig. S4, available at *Rheumatology Advances in Practice* online). The most common symptom was arthralgia [with a prevalence of 41% (95% CI 10, 77)] rather than fatigue [with a prevalence of 39% (95% CI 21, 57)] in these patients. Notwithstanding these limitations, our review provides an overview of long COVID in ARD patients.

## Conclusions

Characteristics of long COVID in ARD patients are basically similar to those in the general population despite a higher prevalence and more of them suffering from arthralgia and pain. In ARD patients, severe COVID-19 infection is a potential risk factor for long COVID. Vaccination and timely treatment for COVID-19 in ARD patients could probably reduce the incidence of long COVID and should be recommended by rheumatologists.

## Supplementary Material

rkae027_Supplementary_Data

## Data Availability

The data underlying this article will be shared upon reasonable request to the corresponding author.
