# Supplementary material for: Characteristics of long COVID in patients with autoimmune rheumatic diseases: a systematic review and meta-analysis
Source: Rheumatol Adv Pract. 2024 Mar 2;8(2):rkae027. doi: 10.1093/rap/rkae027 (PMC10980592; doi:10.1093/rap/rkae027)
Supplement: rkae027_Supplementary_Data [file rkae027_supplementary_data.docx]

**Supplementary Table S1.** Search strategies.

| **PubMed** |
| --- |
| #1 Post-Acute COVID-19 Syndrome[MeSH] OR Post-Acute COVID*[tiab] OR Long Haul COVID*[tiab] OR Post Acute COVID*[tiab] OR Long COVID*[tiab] OR Post-Acute Sequelae of SARS*[tiab] OR Post Acute Sequelae of SARS*[tiab] OR Post-COVID*[tiab] OR Post COVID*[tiab] OR Long-Haul COVID*[tiab]  #2 Connective Tissue Diseases[MeSH] OR Connective Tissue*[tiab]  #3 Rheumatic Diseases[MeSH] OR Rheuma*[tiab]  #4 systemic lupus*[tiab] OR Libman Sacks*[tiab]  #5 Arthritis[MeSH] OR arthri*[tiab] OR Polyarthri*[tiab]  #6 Sjogren's Syndrome[MeSH] OR Sjogren*[tiab] OR Sicca*[tiab]  #7 Spondylarthropathies[MeSH] OR Spondyloarthro*[tiab] OR Spondylarthro*[tiab]  #8 Systemic Sclero*[tiab] OR Localized Sclero*[tiab] OR Morphea*[tiab] OR Linear Sclero*[tiab]  #9 Myositis[MeSH] OR Myosit*[tiab] OR Inflammatory Muscle*[tiab] OR Inflammatory Myopath*[tiab] OR Idiopathic Inflammatory Myo*[tiab]  #10 Hughes Syndrome*[tiab] OR Antiphospholipid*[tiab] OR Anti-Phospholipid*[tiab] OR Anti Phospholipid*[tiab]  #11 Vasculitis[MeSH] OR Vasculiti*[tiab] OR Angiiti*[tiab]  #12 Cryoglobulinemia[MeSH] OR Cryoglobulin*[tiab]  #13 Still's Disease, Adult-Onset[MeSH] OR Adult-Onset Still*[tiab] OR Adult Onset Still*[tiab]  #14 Fibromy*[tiab] OR Fibrositi*[tiab] OR Secondary Fibromy*[tiab]  #15 #1 AND (#2 OR #3 OR #4 OR #5 OR #6 OR #7 OR #8 OR #9 OR #10 OR #11 OR #12 OR #13 OR #14) |
| **EMBASE** |
| #1 'long covid'/exp OR 'chronic covid*':ab,ti,kw OR 'covid long-haul*':ab,ti,kw OR 'covid-19 long-haul*':ab,ti,kw OR 'long haul covid*':ab,ti,kw OR 'long hauler covid*':ab,ti,kw OR 'post covid*':ab,ti,kw OR 'post-acute covid*':ab,ti,kw  #2 ‘connective tissue disease’/exp OR ‘connective tissue*’:ab,ti,kw  #3 ‘rheumatic disease’/exp OR ‘rheuma*’:ab,ti,kw  #4 ‘systemic lupus*’:ab,ti,kw OR ‘lupus*’:ab,ti,kw  #5 ‘arthri’:ab,ti,kw OR ‘joint inflammat*’:ab,ti,kw  #6 ‘Sjogren*’:ab,ti,kw OR ‘sicca*’:ab,ti,kw  #7 ‘spondylarthropathy’/exp OR ‘spondyl*’:ab,ti,kw  #8 ‘scleroderm*’:ab,ti,kw OR ’systemic sclera*’:ab,ti,kw  #9 ‘myositis’/exp OR ‘idiopathic inflammatory myo*’:ab,ti,kw OR ‘inflammatory myo*’:ab,ti,kw OR ‘myositis*’:ab,ti,kw  #10 ‘Hughes syndrome*’:ab,ti,kw OR ‘antiphospholipid*’:ab,ti,kw OR ‘primary antiphospholipid*’:ab,ti,kw  #11 ‘vasculitis’/exp OR ‘vasculiti*’:ab,ti,kw OR ‘angiiti*’:ab,ti,kw  #12 ‘cryoglobulinemia’/exp OR ‘cryoglobulin*’:ab,ti,kw  #13 ‘adult-Onset Still*’:ab,ti,kw  #14 ‘fibromyalgia’/exp OR ‘fibrositi*’:ab,ti,kw  #15 #1 AND (#2 OR #3 OR #4 OR #5 OR #6 OR #7 OR #8 OR #9 OR #10 OR #11 OR #12 OR #13 OR #14) AND [2020-2021]/py |

**Supplementary Table S2.** Study characteristics.

| Study  (reference number) | Country | Study design | COVID-19 care | Sample size | Proportion of female | Age (mean±SD, median [IQR]) (years) | Race | Type of rheumatic diseases | Outcome ascertainment | Definition of long COVID | Symptom duration (mean/median (IQR)) (days) |
| --- | --- | --- | --- | --- | --- | --- | --- | --- | --- | --- | --- |
| Aronova et al. (8) | Russia | Cross-sectional | Outpatients and inpatients | 23 | 91% | 52±18 | N.A. | RA 100% | Questionnaire | WHO | ≧56 |
| Barbhaiya et al. (9) | USA | Cross-sectional | Outpatients and inpatients | 254 | 80% | 56±15 | White 91% Hispanic 8% | Inflammatory arthritis 24%, SpA 13%, OA 9% | Web-based survey | Persistent symptoms | ≧84 |
| Batıbay et al. (10) | Turkey | Cross-sectional | Outpatients and inpatients | 53 | 72% | 49±14 | N.A. | SpA 30%, RA 19%, CTD 19% | Clinical evaluation | NICE guideline | >84 |
| Brito-Zerón et al. (11) | The Big Data Sjögren Consortium | Prospective cohort | Outpatients and inpatients | 132 | 96% | 55±14 | N.A. | Primary Sjogren's syndrome 100% | Clinical evaluation | NICE guideline | >84 |
| Büyükşireci et al. (12) | Ireland | Cross-sectional | Outpatients and inpatients | 77 | 100% | 47±9 | N.A. | Primary FM 100% | Questionnaire | N.A. | 224 (140, 252) |
| Cox et al. (13) | UK | Prospective cohort | Outpatients and inpatients | 45 | 80% | 59 | White 91% BAME 9% | N.A. | Web-based survey | N.A. | ≧28 |
| Fernández‑de‑las‑Peñas et al. (14) | Spain | Cross-sectional | Outpatients and inpatients | 31 | N.A. | N.A. | N.A. | RA 55%, OA 26%, osteoporosis 19% | Telephone interview | N.A. | 235±42 |
| Gomez et al. (15) | Argentina | Retrospective cohort | Outpatients and inpatients | 1915 | 82% | 51 (40, 61) | White 49% | RA 42%, SLE 16% | Clinical evaluation | NICE guideline | >28 |
| Husni et al. (16) | USA | Cross-sectional | Outpatients and inpatients | 264 | 77% | 57±13 | N.A. | SARD 84%, NARD 16% | PROMIS | Worsening of ≥ 5 T score | 84 |
| Iorio et al. (17) | USA | Cross-sectional | Outpatients and inpatients | 174 | 81% | 52±16 | White 80%, black 10%, Asian 5% | RA 40%, SLE 14%, PsA 12% | Online survey | CDC | 25 (11, 160) |
| Leon et al. (18) | Spain | Retrospective cohort | Inpatients | 105 | 64% | 67±15 | N.A. | RA 18%, tendonitis 16%, knee disorder 8%, crystal-induced arthritis 7% | Clinical evaluation | Persistent symptoms | 112-196 |
| Mukhamadieva et al. (19) | Russia | Prospective cohort | Outpatients and inpatients | 55 | 42% | 49 (38, 58) | N.A. | AS 67%, PsA 33% | Clinical evaluation | Exacerbated or new-onset symptoms | N.A. |
| Norgard et al. (20) | Denmark | Retrospective cohort | Inpatients | 417 | 55% | 71 (59, 78) | White>90% | IBD 37%, RA 49%, SpA 12%, PsA 5% | National registry | Hospitalization | ≧28 |
| Rivera et al. (21) | Spain | Cross-sectional | Outpatients and inpatients | 134 | 100% | 52±10 | N.A. | Primary FM 58%, RA 19%, PsA 7%, SLE 6% | Clinical evaluation | Persistent or exacerbate symptoms | 273 |
| Shamsutdinova et al. (22) | Russia | Prospective cohort | Outpatients and inpatients | 105 | 85% | 62 (55, 68) | N.A. | RA 100% | Clinical evaluation | Persistent or new-onset symptoms | 84 |

AS, ankylosing spondylitis; BAME, black, Asian and minority ethnic; CDC, Centers for Disease Control and Prevention; CTD, connective tissue disorder; FM, fibromyalgia; IBD, inflammatory bowel disease; N.A., not available; NARD, non-autoimmune rheumatic disease; PROMIS, Patient Reported Outcomes Measurement Information System; OA, osteoarthritis; PsA, psoriatic arthritis; RA, rheumatoid arthritis; SARD, systemic autoimmune rheumatic disease; SLE, systemic lupus erythematosus; SpA, spondyloarthropathy; WHO, World Health Organization.

**Supplementary Figure S1.** Risk of bias.


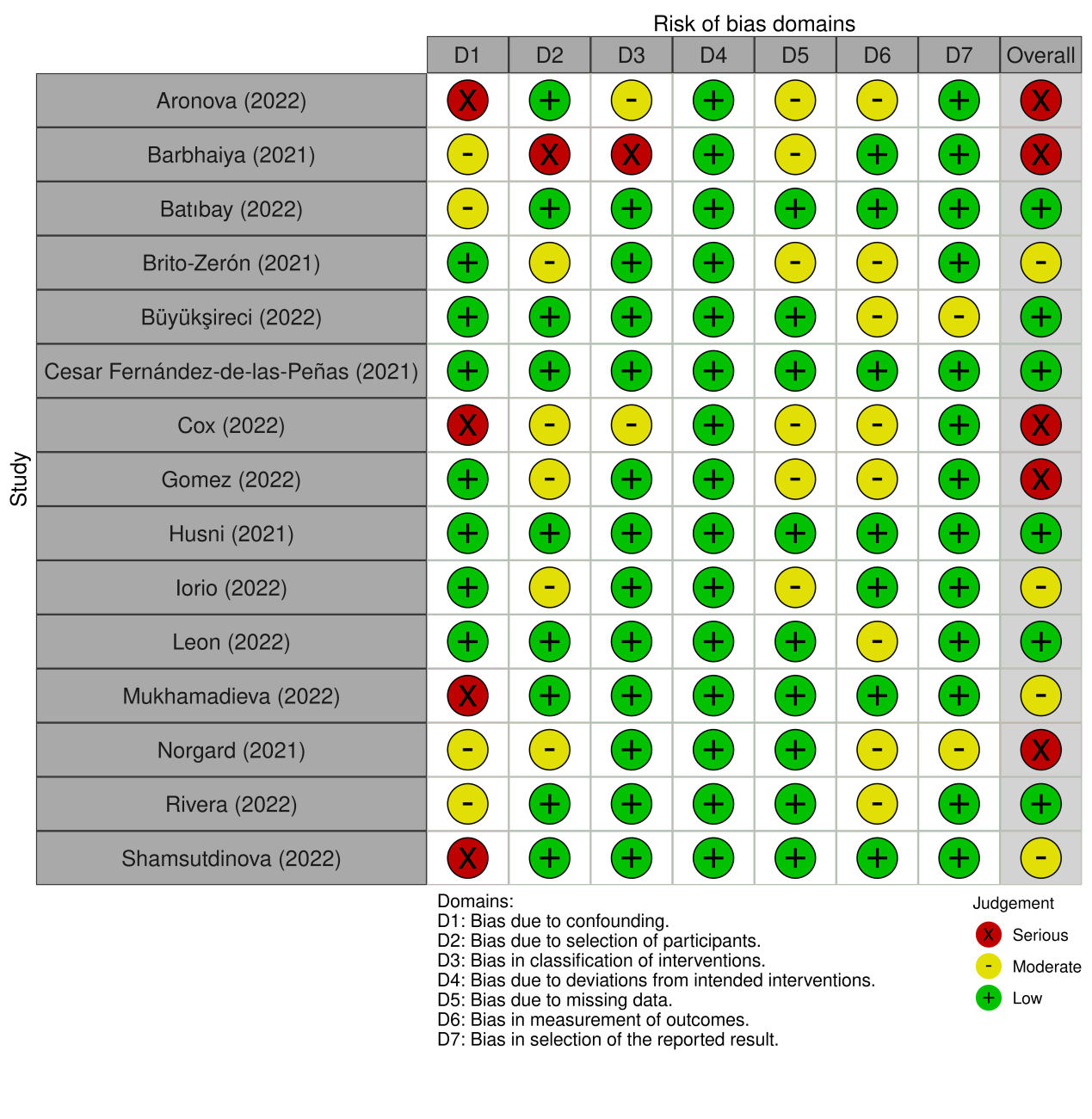


**Supplementary Figure S2.** The funnel plots, as well as Begg’s and Egger’s tests results, of the prevalence of long COVID in rheumatic patients.

**Supplementary Figure S3.** The funnel plots, as well as Begg’s and Egger’s tests results, of the prevalence of long COVID symptoms, such as (a) fatigue, (b) arthralgia, (c) respiratory distress, (d) anosmia, (e) chest discomfort, (f) sleep disturbance, (g) dysgeusia, (h) headache, (i) myalgia, (j) cough, (k) cognitive complaints, (l) depression, (m) fever, (n) sore throat, and (o) nausea, in rheumatic patients.

(a)

(b)

(c)

(d)

(e)

(f)

(g)

(h)

(i)

(j)

(k)

(l)

(m)

(n)

(o)

**Supplementary Figure S4.** The prevalence of long COVID symptoms in rheumatic patients after exclusion

of studies with a serious risk of bias. The black squares represent the summary effect estimates.
